# Association of social support with negative emotions among Chinese adolescents during Omicron-related lockdown of Shenzhen City: The roles of rumination and sleep quality

**DOI:** 10.3389/fpsyt.2022.957382

**Published:** 2022-08-15

**Authors:** Tianyou Guo, Zhihao Zhang, Alyx Taylor, Daniel L. Hall, Albert S. Yeung, Arthur F. Kramer, Liye Zou

**Affiliations:** ^1^Body-Brain-Mind Laboratory, The Shenzhen Humanities & Social Sciences Key Research Bases of the Center for Mental Health, School of Psychology, Shenzhen University, Shenzhen, China; ^2^School of Rehabilitation, Sport and Psychology, AECC University College, Bournemouth, United Kingdom; ^3^Mongan Institute, Harvard Medical School, Boston, MA, United States; ^4^Massachusetts General Hospital, Boston, MA, United States; ^5^Depression Clinical and Research Program, Massachusetts General Hospital, Boston, MA, United States; ^6^Department of Psychology, Center for Cognitive and Brain Health, Northeastern University, Boston, MA, United States; ^7^Beckman Institute, University of Illinois at Urbana-Champaign, Champaign, IL, United States

**Keywords:** depression, anxiety, rumination, sleep quality, social support

## Abstract

**Objective:**

Adolescents are likely to suffer from negative emotions such as depression and anxiety due to the rapid development of biological, cognitive and social changes. Previous studies have indicated possible risk (rumination) and protective (good social support and high sleep quality) factors for depression and anxiety among this age group. The present study is the first to investigate the association between social support and negative emotions during the Outbreak of Omicron variant, on this basis, to further determine the mediating role of rumination and sleep quality on this link.

**Method:**

A total of 1,065 Chinese middle- and high-school students (51.5% female, *M*_*age*_ = 13.80, *SD* = 1.20) completed a psychosocial battery, including the Social Support Rating Scale (SSRS), the Pittsburgh Sleep Quality Index (PSQI), the Ruminative Responses Scale (RRS), the Depression Anxiety Stress Scale (DASS). Serial multiple mediation analysis was conducted using PROCESS macro based on SPSS.

**Results:**

Social support, rumination, and sleep quality were significantly negatively correlated with negative emotional states (Ps < 0.05). Further, rumination and sleep quality were found to partially mediate the relationship between social support and negative emotional states.

**Conclusions:**

For early detection and prevention of depression and anxiety, providing sufficient social support is necessary for adolescents, because rumination and sleep problems are reported during stressful periods, such as the COVID-19 pandemic.

## Introduction

Negative emotions such as depression and anxiety are common when facing a life-threatening event like the COVID-19 pandemic (1, 2). These negative emotions can be more severe among individuals who have been forced to quarantine at home or other places such as a hotel during city lockdown. Shenzhen is one of the most economically developed, densely populated cities in China recently faced its 3rd city-wide lockdown. All residents were required to stay at home for more than a week, including students for whom online classes were developed. The youth experienced unprecedented isolation from their peers and teachers, which has substantial impacts on emotions (COVID-related increases in psychological distress) among high school students (3, 4). Thus, there is an urgent need to identify factors that buffer against the negative effects of social isolation on emotions among the adolescent population during the outbreak of the Omicron variant, using high school students in Shenzhen as an exemplary sample.

Research has revealed key predictors of negative emotional states such as social support, sleep quality, rumination and allostatic load (5–8). Social support refers to the perception of being cared for, esteemed and valued by others (9). This has been identified as one of the strongest protective factors against negative emotional states (10). Studies have consistently shown that social support acts as a buffer against psychological distress such as depression and anxiety caused by stressful life events (7, 11, 12). Specifically, social support comes into play in dealing with depression and anxiety during times of stress, especially in adolescents (13, 14). There are many sources of social support for Chinese adolescents, primarily family (e.g., parents) and school (e.g., teachers and classmates). The buffering effect model, a major social support theory, suggests that social support is related to the subjective assessment of people under stress (15).

Rumination is a type of unpleasant experience that individuals present repetitive and passive thinking about the same event or thing (16). Several studies suggested that rumination appears to be a transdiagnostic factor in depression and anxiety, a mechanism responsible for the high degree of comorbidity among certain mental disorders, as it amplifies and prolongs negative emotional states (5, 17–19). Other empirical studies showed that empirical studies showed that rumination is significantly associated with low social support (20, 21). Social support is known to protect individuals under stress from negative emotional states and maladaptive response (e.g., rumination). Thus, the mediating role of rumination on relationship between low social support and negative emotions during the outbreak of Omicron variant should be investigated.

Among the many pathways, sleep appears to have a particularly important role (22). A relatively large, and growing, body of literature has shown that sleep quality is a key predictor of both physical and mental health (23–26). Specifically, poor sleep quality has been linked to higher risk of depression and anxiety (27, 28). There is robust evidence for inflammation as a biological pathway linking sleep quality and depression (29, 30). In addition, regarding the positive impact of social support on sleep, evolutionary psychology explains that close associates protect individuals from danger during the sleep phase (31). Some studies have supported this hypothesis that higher levels of social support are associated with better sleep quality (22, 32). Therefore, it is crucial to explore the specific role of sleep quality between social support and negative emotional states to provide more effective methods for the intervention of adolescents trapped in psychological distress. Furthermore, the COVID-19 outbreak as a serious stressful life event was associated with higher levels of rumination, leading to poor sleep quality (33). Taken together, sleep quality and rumination may act as serial mediators of the relationship between social support and negative emotional states. However, the empirical evidence in this field is not sufficient and needs to be explored urgently.

To our knowledge, no previous studies have explored the associations among of social support, sleep quality, rumination, and negative emotional states. Therefore, the aim of the present study was to propose a serial multiple mediation model to examine the mediating effects of sleep quality and rumination between social support and negative emotional states. Accordingly, we hypothesized that: (1) negative emotional states would be negatively correlated with social support and sleep quality, and positively correlated with rumination; (2) rumination and sleep quality play mediating roles in the relationship between social support and negative emotional states.

## Methods

### Study participants

Four high schools (HS) from Shenzhen, China, were selected from March to April 2022 using convenience sampling. Students aged 12–18 years old from the selected schools were recruited. Permission for this in-school survey was obtained before the investigation from schools, legal guardians, and students. Participants were asked to complete an electronic questionnaire through a platform (Wenjuanxing-called Questionnaire Star), with the background, aim, and anonymity of the study being presented at the top of the questionnaire. A total of 1,179 HS and junior high school (JHS) students volunteered to take part in this study. After all the participants had completed the study, the data with and unacceptably short duration for response (<3 min to complete the e-questionnaire), or failing the lie detector quiz (*n* = 114) were removed, and valid answers were obtained from 1,065 participants (548 females, 517 males, *M*_*age*_ = 13.8, *SD* = 1.2). This study protocol (PN-2020-041) was approved by the ethical committee of Shenzhen University before data collection.

### Measures

Negative emotional states were measured using the Chinese version of Depression Anxiety Stress Scale [DASS; (34, 35)], which is a self-report questionnaire in the present study. This questionnaire includes 21 items, with each rated on a 4-point scale: Zero (Did not apply to me at all) to three (Applied to me most of the time). Cronbach's alpha of this questionnaire in the present study was 0.88.

The Chinese version of the Pittsburgh Sleep Quality Index (PSQI) was used to measure sleep quality in the sample (36, 37). This scale includes 19 items assessing seven dimensions of sleep quality: subjective sleep quality, sleep latency, sleep duration, habitual sleep efficiency, sleep disturbances, use of sleep medication, and daytime dysfunction. Specifically, a greater total score indicates worse subjective sleep quality. Cronbach's alpha of this questionnaire in the present study was 0.85.

The Ruminative Responses Scale (RRS) is a widely used self-administered measure of rumination (38, 39). It consists of 22 items and three domains of rumination (symptom rumination, brooding, and reflective pondering), with each rated on a 4-point scale (1–4). The total score ranges from 22 to 88, with higher score indicating more severe rumination. Cronbach's alpha of this questionnaire in the present study was 0.88.

Social support was assessed using the Social Support Rating Scale [SSRS; (40)]. The SSRS is a 10-item self-report instrument that measures 3 domains of social support (subjective social support, objective social support, and the utilization of social support). The global score for this scale can be obtained by adding up the scores of each item (range 12–66); higher scores indicate greater level of social support. Cronbach's alpha of this questionnaire in the present study was 0.95.

Sedentary time was also measured using a part of the International Physical Activity Questionnaire-short form [IPAQ-SF; (41, 42)]. A single-item question was used to collect the duration of sedentary behaviors of participants. Specifically, participants were asked to fill out the information of “— hours per day” and then “— minutes per day” following “During the last seven days, how much time did you spend sitting on a week day.” Daily duration in hours was converted into minutes. Given that sedentary behavior was negatively correlated with social support (*r* = −0.12, *p* < 0.001) and sleep quality (*r* = −0.16, *p* < 0.001) but it is positively linked to negative emotion (*r* = 0.16, *p* < 0.001), it was considered as a covariate in this study.

### Statistical analysis

Data were analyzed with SPSS 22.0 and PROCESS which is a widely used a macro program for SPSS to analyze mediation and moderation models (43). Descriptive analyses were first conducted of socio-demographic characteristics and the calculation of means and standard deviation (*SD*). Pearson's correlation was used to examine the association between each two continuous variables, after controlling for several co-variables including gender, age and BMI (Body mass index). To understand the mechanism of social support, sleep quality, rumination and negative emotional states, hypothesized mediation analyses were examined using the PROCESS macro. Multiple mediation analyses were based on bootstrapping (5,000 bootstrap samples) with 95% confidence intervals (CI). An effect was considered as significant at the 0.05 level if the 95% bias-corrected bootstrap CI of the mediation effect does not contain zero. Gender, age and BMI were considered as covariates in the model. *P*-values < 0.05 were considered statistically significant when a two-tailed test was used.

## Results

### Descriptive analyses

Results for gender difference on socio-demographic and anthropometric variables are presented in Table 1. A significant gender difference was observed on age, BMI, PSQI, and DASS. Of note, compared with male counterparts, female participants demonstrated significantly lower sleep quality and higher negative emotional states.

**Table 1:** Gender difference on sociodemographic and anthropometric variables.

**Variables**	**Total (1,065)**	**Male (517)**	**Female (548)**		
	***M ±SD***	***M ±SD***	***M ±SD***	***t***	***p***
Age (years)	13.77 ± 1.23	13.89 ± 1.27	13.66 ± 1.19	2.94^**^	0.003
BMI (kg/m^2^)	19.99 ± 3.32	20.73 ± 3.65	19.39 ± 2.80	7.15^***^	<0.001
Sedentary time (minutes)	446.65 ± 169.443	431.82 ± 166.36	460.63 ± 171.28	−2.784^**^	0.005
SSRS	34.61 ± 7.03	34.62 ± 7.15	3,460 ± 6.93	0.06	0.952
PSQI	5.15 ± 3.17	4.89 ± 3.15	5.39 ± 3.17	−2.55^*^	0.011
RRS	22.28 ± 6.27	22.07 ± 6.30	22.49 ± 6.25	−1.087	0.277
DASS	12.26 ± 9.40	11.41 ± 9.20	13.07 ± 9.53	−2.879^**^	0.004

*SSRS, Social Support Rating Scale; RRS, Ruminative Responses Scale; PSQI, Pittsburgh Sleep Quality Index; DASS, Depression Anxiety Stress Scale*.

* *p < 0.05,*

** *p < 0.01,*

*** *p < 0.001*.

### Correlation analyses

Correlations for the key study variables under the control of gender, age and BMI are presented in Table 2. Social support and sleep quality were both negatively correlated with rumination and negative emotional states. In addition, rumination was significantly and positively correlated with negative emotional states.

**Table 2:** Correlations of all tested variables.

**Variables**	**SSRS**	**PSQI**	**RRS**	**DASS (total score)**
**SSRS**				
PSQI	−0.175^***^			
RRS	−0.125^***^	0.117^***^		
DASS (total score)	−0.321^***^	0.248^***^	0.320^***^	

*** *p < 0.001*.

*SSRS, Social Support Rating Scale; RRS, Ruminative Responses Scale; PSQI, Pittsburgh Sleep Quality Index; DASS, Depression Anxiety Stress Scale*.

### Multiple mediation model

**Figure 1:**
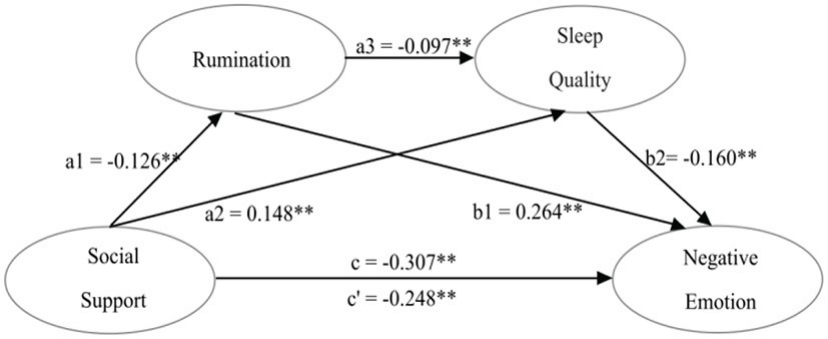
The mediation model. **p < 0.01.

Multiple mediation analysis was conducted in PROCESS to explore the mediators of social support and negative emotional states among teenagers, with gender, age, BMI, and sedentary time as covariates. The total, direct, and indirect effects are listed in Table 3; Figure 1, the bias-corrected 95% CI for all six paths did not include zero. The analysis revealed that the total and direct effect of social support on negative emotional states were statistically significant, indicating that high level of social support was associated with less negative emotional states. Social support was found to indirectly affect negative emotional states through three negative significant mediation pathways: (i) rumination (Standardized Effect = −0.0336, 95%CI [−0.0709, −0.0222]); (ii) sleep quality (Standardized Effect = −0.0237, 95%CI [−0.0537, −0.0149]); (iii) rumination and sleep quality (Standardized Effect = −0.0020, 95%CI [−0.0054, −0.0008]). Specifically, results showed that the negative association between high level of social support and less negative emotional states was mediated by less rumination and higher level of sleep quality.

**Table 3:** Mediation modeling results.

**Path**	**Standardized effect**	**SE**	**LLCI**	**ULCI**
Total effect	−0.3072	0.0386	−0.4863	−0.3349
Direct effect	−0.2478	0.0370	−0.4039	−0.2587
Total indirect effects	−0.0593	0.0166	−0.1139	−0.0491
Indirect 1	−0.0336	0.0124	−0.0709	−0.0222
Indirect 2	−0.0237	0.0099	−0.0537	−0.0149
Indirect 3	−0.0020	0.0012	−0.0054	−0.0008

*SE, standard error; LLCI and ULCI, lower level and upper level of the bias-corrected 95% bootstrap confidence interval*.

## Discussion

The aim of the present study was to examine the possible links between social support, rumination, sleep quality and negative emotional states (e.g., depression, anxiety, and stress). Consistent with previous studies (44–47), our results showed negative emotional states to be significantly negatively correlated with social support and sleep quality, and positively correlated with rumination which also concur with our theoretical hypothesis. Furthermore, the results of this study increase the understanding of the underlying psychological mechanism regarding the link between social support and negative emotional states. Based on the serial multiple mediation model, the present study explained how social support contributes to the reduction of negative emotional states.

The allostatic load model suggests that when repetitive allostatic responses are activated, the body undergoes a cumulative “wear and tear” which may become an important trigger for negative emotional states such as depression and anxiety (48). Specifically, prolonged activation of the HPA (hypothalamic–pituitary–adrenal) axis can indeed trigger pathological changes. However, anxiety and/or depression can develop in response to stress before such physical changes. The stress/allostatic load—poor mental health—poor physical health relationship is more cyclical than linear and can be triggered at different points. For this population of healthy adolescents, the risk of entry into the cycle is stress leading to mental health problems. The key point is that the current results demonstrate the direct and indirect paths by which social support can be a protective factor against the risk of anxiety and/or depression being triggered by the stressful situation. Thus, good social support helps to prevent the cycle of prolonged stress—poor mental health—poor physical health. Previous studies have shown that social support is one of the most important protective factors for allostatic load leading to negative emotional states (49, 50). Rumination has been shown to be a precursor to mental disorders such as depression (51), and there are empirical studies suggesting that post-stress rumination may be a predictor of HPA axis non-habituation, an important physiological mechanism leading to depression (52). In line with our study, social support predicted negative emotional states indirectly through rumination. In the face of stressful events in life, perceived social support helps individuals to relieve themselves from repetitive, negative, unnecessary rumination, thereby reducing the production of negative emotional states (possibly due to habituation of the HPA axis).

The results of the multiple mediation model further showed that the sleep quality related to rumination was another significant mediator of the association between social support and negative emotional states, extending previous findings about this relationship among older adults (53). Specifically, adolescents with higher perceived social support also show a higher level of sleep quality, which, in turn, led to less negative emotional states during COVID-19 outbreak. Consistent with the results of the present study, a review found sleep as a potential mechanism linking social support and mental health (54). Physiological changes are critical in the development of depression and anxiety, and the allostatic load model highlights the positive effects of high social support and high sleep quality in the physiological mechanisms associated with negative emotional states. In particular, poor sleep quality/quantity contributes to allostatic load that makes the body more vulnerable to the impacts of stressors of life, enhancing the disruptive effects of stressful life events on emotional states (55). To the best of our knowledge, few studies have investigated the mediating effect of sleep quality on social support and negative emotional states caused by major life stress events such as COVID-19 outbreak. In this case, it's a new perspective on improving social support, which improves sleep quality and ultimately reduces stress, anxiety, and depression.

Furthermore, the most critical finding of the present study was that social support exerts an influence on negative emotional states is mediated by rumination and sleep quality among Chinese adolescents. Specifically, the pathway is social support → rumination → sleep quality → negative emotional state. COVID-19 outbreak is a very serious life stress event, which is likely to cause negative emotional states such as depression and anxiety (56). We hold the opinion that under the same conditions of stress events, individuals with a high level of social support tend to perceive a lower level of stress or be more effective at coping with stress (57), so they spend less time and mental effort ruminating about the epidemic and related life stress events, which buffers against the decline of sleep quality caused by stressful events, and better sleep protects against depression and anxiety (likely through various psychoneuroimmunological processes not assessed in this survey study). Social support as the protective factor of negative emotions should be taken into account at school settings because it could help researchers better understand the COVID-induced effect; Psychological First Aid and Skills for Psychological Recovery are recommended to facilitate inter-person interaction during isolation and are the adapted methods to respond to COVID-specific needs for what may be a long-term isolation and post isolation (58). The current study represents an important part of understanding the mechanisms underlying the link between social support and negative emotional states, and complement the allostatic load model with crucial psychological evidence.

## Limitations

Several limitations of this study should be mentioned. Firstly, allostatic load is a well-researched theory that has been very influential in terms of the generation and development of negative emotions. However, due to the epidemic, this study could not collect the relevant physiological data of the allostatic load model. Secondly, this study did not rule out the existence of other potential mediating variables. Thirdly, social support was measured by self-report of perceived support, which could be influenced by one's state of emotions. Lastly, a cross-sectional examination of the mediation model was performed, which limits conclusions about causality.

## Data availability statement

The original contributions presented in the study are included in the article/supplementary materials, further inquiries can be directed to the corresponding authors.

## Ethics statement

The studies involving human participants were reviewed and approved by the Ethical Committee of Shenzhen University. Written informed consent from the participants' legal guardian/next of kin was not required to participate in this study in accordance with the national legislation and the institutional requirements.

## Author contributions

LZ and TG participated in the design of the study and the manuscript editing. ZZ and TG participated in the manuscript drafting and data reduction/analysis. AT and DH contributed to data reduction/analysis and manuscript editing. AY and AK contributed to the interpretation of results and manuscript editing. All authors have approved the final version of the manuscript and agreed with the order of presentation of the authors.

## Funding

This project was supported by National Natural Science Foundation of China (31871115), Start-up Research Grant of Shenzhen University (20200807163056003) and Start-Up Research Grant (Peacock Plan: 20191105534C).

## Conflict of interest

The authors declare that the research was conducted in the absence of any commercial or financial relationships that could be construed as a potential conflict of interest.

## Publisher's note

All claims expressed in this article are solely those of the authors and do not necessarily represent those of their affiliated organizations, or those of the publisher, the editors and the reviewers. Any product that may be evaluated in this article, or claim that may be made by its manufacturer, is not guaranteed or endorsed by the publisher.
